# Extracting Teeth

**Published:** 1891-05

**Authors:** W. H. Sutton

**Affiliations:** Magog, Que.


ARTICLE VI.

### EXTRACTING TEETH.

BY W. H. SUTTON, L. D. S., MAGOG, QUE.

While condemning, as a rule, the extraction of teeth which prevailed to such an extent in larger cities, it is not possible that their extraction will ever become out of place, and it is important to make this matter as easy as possible, with as little pain to the patient and as little labor to the operator. Some of our oldest operators still adhere to the use of the key and the elevators, and in spite of our objections to them, very skillful operations have been performed by them. My late father for nearly half a century used these instruments, and he never had an accident; and I believe there are enough operators to-day using the elevator to make it worth the while of the depots to add new forms and keep old forms in stock.

In my practice in the country with my late father for over ten years, I used only a few forceps for all cases, as I found that I could accomplish all I needed with four or five.

1. The bayonet forcep.—This is one of the most important of our extracting instruments, and can be made to do good work as well in the lower jaw as in the upper. It should not be too long in the handle, and should have both ends of handle bent or curved so as to make it easy of adaptation to the palm of the hand.

If the patient is placed on a stool, or if the dentist stands on one, this forcep can be used for roots of lower bicuspids and molars; sometimes, too, when the jaws are partially closed by inflammation. When the *denta sapientiæ* are developing, the easiest way to reach them often is by the bayonet forcep, when by slow movement they can be extracted.

2. Many dentists find the ordinary forcep badly adapted for the hand, and it is a common thing to see dentists placing a ball of cotton in the hollow of the hand to get a solid grip of the forcep handle. I am bothered with this difficulty myself, and I beg respectfully to present my means of overcoming the trouble. I place on one of the handles a rubber bulb, which adapts itself to the hollow of the hand and gives me a steady hold, and takes away the spring which is found in many handles of American forceps.

3. It is often a useful thing to place a cork gag between the teeth before extracting a difficult lower tooth. The movement of the lower jaw is frequently so pliable that it is not easy to control it, and a steady gag assists the operator, and prevents accidents by striking the upper teeth in the last action of extraction. It seems to be a relief to the patient, as he has not that sense of fracturing a part which so many people feel in having difficult teeth extracted.

4. After extracting abscessed roots and teeth associated with large gum-boils, it is a good precaution to rinse the mouth immediately with a hot solution of water and salt, or water and carbolic acid; and in deep-seated abscesses it is wise to syringe the sockets with peroxide of hydrogen, to cleanse the pus away. The instruments should be thoroughly disinfected by cleansing them in a five per cent. carbolic acid solution. There is a great danger of infection from dirty forceps. Blood poisoning may occur from unclean instruments.—*Dominion Dental Journal.*

